# Orthostatic hypotension and complaints: not a clear-cut relation

**DOI:** 10.1007/s10286-026-01222-3

**Published:** 2026-06-16

**Authors:** Boriana S. Gagaouzova, Ineke A. van Rossum, Fabian I. Kerkhof, Roland D. Thijs, J. Gert van Dijk

**Affiliations:** 1 Department of Neurology, Leiden University Medical Centre, Albinusdreef 2, 2333 ZA Leiden, The Netherlands; 2 Stichting Epilepsie Instellingen Nederland (SEIN), Achterweg 3, 2103 SW Heemstede, The Netherlands; 3 UCL Queen Square Institute of Neurology, University College London, London, WC1N 3BG UK; 4 Department of Neurology, Leiden University Medical Centre, PO Box 9600, 2300 RC Leiden, The Netherlands

**Keywords:** Orthostatic hypotension, Symptoms, Log-ratio, Pathophysiology

## Abstract

**Purpose:**

Previous research regarding the association between blood pressure (BP) fall and symptoms yielded inconsistent results in classical orthostatic hypotension (cOH), with some studies proposing specific cut-off values and others reporting only weak or absent correlations. This study examined clinical cOH symptoms in relation to changes in BP and the haemodynamic parameters determining BP.

**Methods:**

We retrospectively analysed 77 tilt test records showing cOH and selected 40 with and 37 without complaint recognition during the test. We compared absolute values of haemodynamic parameters as well as the differences and ratios compared to supine baseline at three different times during tilt. We also explored relationships between relative haemodynamic changes and symptoms with the log-ratio method.

**Results:**

There was a larger blood pressure fall in the group with complaint recognition compared (49.2 vs 37.3, *p* = 0.043). The log-ratio analysis showed less total peripheral resistance increase and a larger blood pressure decrease in the symptomatic group.

**Conclusion:**

Recognised complaints were related to a larger BP fall, while in previous studies complaints were either not related to BP or to minimum BP. These contrasts suggest that the pathophysiology of orthostatic complaints is complex. The chain of events from low BP to awareness of complaints contains multiple links, of which cerebral perfusion is probably a critical one. We suggest that the pathway contains so much variability that we should not expect the relation between low blood pressure and complaints in cOH to be simple.

## Introduction

Classical orthostatic hypotension (cOH) is defined as a fall of at least 20 mmHg of systolic blood pressure (BP) or 10 mmHg of diastolic BP in the first 3 min after standing up or after head-up tilt [1, 2]. cOH can be one of the first signs in neurodegenerative diseases and is associated with various symptoms, ranging from light-headedness to cognitive slowing and loss of consciousness, i.e. syncope [3–7]. cOH can negatively impact quality of life, due to recurrent falls with injuries, cognitive impairment, increased risk of cardiovascular conditions and increased mortality [4, 7–9], and can also contribute to additional, possibly preventable, medical costs.

The recognition of complaints as being due to cOH by medical practitioners is crucial for the diagnosis and management of cOH. However, the attribution of symptoms to cOH poses multiple clinical challenges. Of note, the published definition of cOH comprises a measurement, i.e. a threshold of an upright BP fall, but does not mention signs or symptoms [2, 10].

Problems with attributing symptoms to cOH start with a large variability in the nature of reported symptoms, which include light-headedness and syncope, but also fatigue, malaise, trouble concentrating, visual disturbances, reduced attention, pain in the shoulder, chest or neck areas, and a reduced capacity for exercise [11]. One previous study on the relation between complaints and BP parameters reported no correlation between the magnitude of the BP fall and the presence of complaints, with about one third of patients with a considerable systolic BP (SBP) fall of 60 mmHg remaining completely asymptomatic [12]. This lack of correlation between symptoms and BP appeared to have been solved when Palma et al. reported a strong relation between mean systolic BP and orthostatic symptoms after 3 min of standing in participants with Parkinson’s disease (PD), resulting in a high sensitivity and specificity for a mean arterial pressure (MAP) threshold of 75 mmHg [11]. This finding suggested that the occurrence of symptoms was tied to low SBP itself rather than to the magnitude of the BP fall. Unfortunately, no correlation was later found in a study by Freeman et al. on patients with various causes of OH, neither for the BP fall nor for absolute BP [13].

As these previous studies showed divergent findings regarding the relation between absolute BP or BP fall and the occurrence of symptoms in cOH, we investigated absolute BP, BP changes and the presence or absence of recognised complaints during a tilt test in a cOH population. We also used the ‘log-ratio method’ that allows a quantitative comparison of relative effects on MAP of three haemodynamic parameters: heart rate (HR), stroke volume (SV) and total peripheral resistance (TPR) [14].

## Methods

### Study population

The study population was described previously (Gagaouzova et al. 2025). In short, we collected reports of tilt table test (TTT) performed at the department of Clinical Neurophysiology of the Leiden University Medical Centre in the period 2010–July 2022, resulting in a group with cOH and a control group without cOH or other haemodynamic tilt test abnormalities. The present study focuses on cOH data only [15]. The Leiden centre accepts regional and national referrals for autonomic disorders. Data were collected in two steps: the first regarded inspection of TTT reports and clinical data, and the second consisted of gathering haemodynamic data on a 1-s timescale.

The first step started with selection of cOH TTT reports that included reason for referral, a graph showing BP and HR of the entire TTT and remarks describing events (‘tilt up’, ‘syncope’, etc.), including whether complaints and symptom recognition occurred; the latter is considered an essential part of any tilt test in our laboratory. We assessed the presence of cOH according to published guidelines [3–6] and judged quality of BP and HR data visually, based on missing data, artefacts, extra systolic beats and the presence of a pacemaker. Quality was qualified as good, moderate or poor, with pacemaker presence always resulting in ‘poor’. Inclusion required cOH as the only haemodynamic conclusion of the tilt test, i.e. additional diagnoses such as vasovagal syncope, POTS, etc., were reason for exclusion, in addition to poor or moderate quality.

Clinical data included age, sex, reason for TTT and medication at the time of TTT. The latest known diagnosis was documented, using data obtained well after TTT when available. We classified medication with a possible BP effect as increasing or decreasing BP: BP-increasing drugs were midodrine, fludrocortisone or a combination of both, and BP-decreasing drugs were classified as primarily inhibiting sympathetic activity, primarily causing vasodilatation, other, or a combination; we established previously that medication did not affect the haemodynamic pattern of OH in this population in a major way [15, 16].

To investigate the relation between haemodynamic changes and complaints we initially divided the patient population in three groups based on complaints: group 1 had symptoms recognised as the same as occurring in daily life (complaint recognition), group 2 had no symptoms during the BP fall and group 3 had symptoms during TTT but did not recognise than as the same as those spurring medical attention. We focused on complaint recognition as the purpose of TTT is to provoke an event with complaint recognition and demonstrate the pathophysiological correlate [2]. Patients underwent history taking before TTT and were instructed to report complaints and whether they recognised complaint as identical to those of daily life. The role of unrecognised complaints is not always clear: some may reflect cOH, whereas others, such as foot pain caused by TTT, could be incorrectly attributed to cOH. To address this problem, the three groups were combined twice: we first compared recognised complaints to the combination of no complaints and unrecognised complaints, but also compared complaints, recognised or not, against no complaints.

### Tilt tests and haemodynamic analysis

BP was measured in supine position with a validated automated cuff sphygmomanometer, while continuous values were obtained by finger volume-clamp analysis using Finometer (Finapres) or Nexfin (BMEye). At least one ECG lead was used. Video-EEG monitoring was always used to help detect syncope, artefacts and note complaints. The cOH protocol comprised 10 min of supine rest, followed by head-up tilt to 70° for a duration of up to 20 min; the period could be shortened when patients suffered severe complaints and wished to be tilted back [17].

To analyse events around tilting up, we extracted a maximum of 180s before and 600s after tilting up. These periods were shortened when the condition was changed, e.g. when patients were tilted down because of syncope or complaints within 600 s after tilt-up.

An EEG machine (Nihon Kohden) was used to store all signals including continuous BP. For older data, the continuous BP signal was extracted from EEG records and analysed with Modelflow (TNO, the Netherlands) to derive beat-to-beat data of systolic and diastolic BP (SBP and DBP), MAP, HR, SV and TPR. For later data, Modelflow beat-to-beat analysis results were extracted from the Finapres Nova device (Finapres, the Netherlands). All beat-to-beat data were resampled at 1 Hz to obtain regularly sampled time series to facilitate interindividual analyses [18].

All individual records were interactively inspected with custom Matlab (Mathworks, Natick, USA) software to remove technical artefacts (e.g. calibration signals, changes induced by coughing or excessive movements, and extra systolic beats). Such faulty data were deleted and resulting gaps were linearly interpolated when periods were short and preceded and followed by stable areas of similar magnitude; otherwise, gaps remained present as missing data.

### Reported complaints during TTT

Participants either reported symptoms spontaneously during the TTT or were asked about symptoms by a technician during the test. The severity of symptoms was not assessed systematically. Prior to the test, the technician instructed the participant to report symptoms and to indicate whether he or she recognized them as occurring in daily life as explained above. The timing of symptom onset during the TTT was not recorded. As said, the three groups were combined on two different ways: once as ‘recognised complaints’ versus ‘non-recognised complaints’ plus ‘no complaints’, and once as ‘any complaint’ versus ‘no complaints’.

### Haemodynamic parameters of symptomatic vs asymptomatic groups

We calculated values for MAP, HR, SV and TPR for four periods during TTT: the first represented supine baseline before tilting (‘pre-tilt’), calculated as the average value over 160 s, lasting from 180 to 20 s before tilt-up. The second period was the fourth minute of the TTT (‘tilt-4th’), consistent with the conventional assessment of cOH. Because cOH is defined as a sustained blood pressure drop occurring within the first 3 min after standing, selecting the fourth minute effectively captures this complete 3-min period. The third was a 30-s period at the end of TTT (‘tilt-end’), as blood pressure may continue to decrease throughout the upright period. The cOH protocol stipulated an upright duration of 20 min, only shortened in case of syncope or when patients had severe complaints necessitating tilt back. The fourth period represented a 30-s period surrounding the minimum BP during the upright period (‘tilt-minimum’).

For all four periods, we calculated both differences (Δ) by subtracting values during tilt from pre-tilt values and expressed tilt values as percentages of pre-tilt values. We did so for MAP, HR, SV and TPR.

We calculated log-ratios for the three haemodynamic parameters for the three different periods. We illustrated the course of events using log-ratios for HR, SV and TPR for the two groups.

### Statistical analysis

We used Matlab (Version R2022, Mathworks, Natick, USA) for numerical and statistical analysis. Normality of distributions was tested with the Kolmogorov–Smirnoff test. We used the *t* test to compare the two groups for values of baseline, fourth minute, end of TTT and minimum values. For log-ratios we used the Mann–Whitney test. A threshold value for significance of 0.05 was used. Count data were compared with the ch-square test.

## Results

### Study participant characteristics

Characteristics of cOH participants (*n* = 77) are summarized in Table 1. Forty patients had recognised complaints, 11 unrecognised complaints and 26 had no complaints. Group 1, with recognised complaints, counted 40 patients The combined groups 2 and 3, i.e. those with unrecognised complaints or no complaints, counted 37 patients. Age and sex did not differ between groups.

**Table 1:** Study group characteristics

	1. Recognised complaints	2. Non-recognised complaints	3. No complaints	*P* values 1 vs (2 & 3)	*P* values (1 & 2) vs 3
Age (years, mean ± SD)	66.3 ± 9.7	66.5 ± 8.3	69.2 ± 7.6	0.31	0.16
Male/female (*n*)	30:10	7:4	17:9	0.33	0.52
cOH aetiology (*n*)					
Diabetes	1	1	1	–	–
Drugs	3	1	0	–	–
Multiple system atrophy	6	0	4	–	–
Pure autonomic failure	4	1	3	–	–
Parkinson’s disease	14	3	10	–	–
Other	12	5	8	–	–
BP-increasing drugs (*n*/known)	7/35	0/10	5/26	0.49	0.66
BP-decreasing drugs (*n*/known)	23/35	4/10	16/25	0.81	0.74
Duration of orthostatic complaints (months, mean ± SD)	17.8 ± 11.6	13.1 ± 4.5	15.8 ± 7.2	0.30	0.75

Data are provided for all three groups: those with recognised complaints, those with unrecognised complaints and those without complaints. *P* values are shown for two combinations of the three groups: first for those with recognised complaints versus the other two groups combined, and then for those with any complaint, recognised or not, versus no complaints

Tests for continuous variable regard the *t* test and test for count data regard the chi-square test. Drug use was not known for all participants; ‘known’ indicates the number for whom information was available

The underlying etiology of cOH was described earlier (Gagaouzova et al. 2025) and comprised PD, multiple system atrophy (MSA), pure autonomic failure (PAF), diabetes mellitus (DM) or drugs known to cause orthostatic complaints/OH [15]. Our study population did not include patients with dementia with Lewy bodies or with severe cognitive impairment. For several cases a definite causal diagnosis was unknown, mostly in case of patients seen only once on request of other centres.

### Haemodynamic parameters of symptomatic vs asymptomatic groups

Table 2 shows haemodynamic parameters (SBP, DBP and MAP) for the three original groups (group 1 with complaint recognition, group 2 with unrecognised complaints and group 3 without complaints). We focused on the comparison between those with recognised complaints versus the remainder. There was no significant difference between these two groups for pre-tilt baseline SBP, DBP and MAP. However, BP falls were greater for the group with recognised complaints for all three tilt periods for SBP and MAP and two period for DBP. Likewise, percentile falls indicated larger BP falls for patients with recognised complaints.

**Table 2:** Comparison of haemodynamic parameters between group 1, 2 and 3

Parameter	1. Recognised complaints	2. Non-recognised complaints	3. No complaints	*P* values 1 vs (2 & 3)	*P* values (1 & 2) vs 3
SBP (mmHg)					
Pre-tilt	158.4 ± 35.3	152.6 ± 27.8	151.5 ± 29.0	0.36	0.44
Tilt-4th	109.2 ± 29.5	114.3 ± 18.1	114.5 ± 27.5	0.40	0.52
Tilt-end	103.3 ± 31.5	106.8 ± 19.0	114.5 ± 22.4	0.15	0.08
Tilt-min	95.2 ± 31.2	98.3 ± 19.5	106.3 ± 20.0	0.15	0.07
Δ 4th	49.2 ± 28.6	38.2 ± 16.5	36.9 ± 24.3	0.043*	0.11
Δ end	55.1 ± 33.7	45.7 ± 19.4	36.9 ± 23.5	0.019*	0.013*
Δ min	63.2 ± 33.0	54.3 ± 18.5	45.2 ± 23.3	0.019*	0.013*
% 4th	30.3 ± 14.4	24.5 ± 7.3	23.8 ± 12.4	0.036*	0.098
% end	33.9 ± 17.6	29.3 ± 10.0	23.4 ± 12.6	0.013*	0.006*
% min	39.3 ± 16.8	35.2 ± 9.3	28.8 ± 11.7	0.010*	0.004*
MAP (mmHg)					
Pre-tilt	112.3 ± 25	105.4 ± 19.9	110.1 ± 23.5	0.51	0.91
Tilt-4th	84.4 ± 21.4	87.6 ± 13.7	90.6 ± 22.6	0.27	0.30
Tilt-end	80.2 ± 22.4	83.4 ± 13.7	90.7 ± 18.9	0.07	0.043*
Tilt-min	74.5 ± 22.1	77.1 ± 14	84.9 ± 17.7	0.07	0.033*
Δ 4th	27.9 ± 21.4	17.8 ± 10.3	19.5 ± 15.9	0.036*	0.15
Δ end	32.0 ± 25.0	22.0 ± 12.5	19.4 ± 15.5	0.013*	0.022*
Δ min	37.8 ± 24.8	28.2 ± 12.8	25.3 ± 15.8	0.014*	0.021*
% 4th	23.7 ± 15.7	16.2 ± 7.2	17.3 ± 11.8	0.030*	0.13
% end	27.2 ± 18.6	20.1 ± 9.7	16.7 ± 12	0.008*	0.009*
% min	32.5 ± 17.9	26.2 ± 9.3	22 ± 11.6	0.007*	0.017*
DBP (mmHg)					
Pre-tilt	83.2 ± 17.7	77.6 ± 15.3	83.5 ± 18.7	0.71	0.75
Tilt-4th	70.1 ± 17	71.6 ± 11.1	75.1 ± 20.4	0.32	0.31
Tilt-end	67.4 ± 18.1	69.5 ± 10.8	76.3 ± 17.4	0.07	0.045*
Tilt-min	62.7 ± 18	64.3 ± 9.8	71.8 ± 16.8	0.07	0.034*
Δ 4th	13.2 ± 16.2	6.0 ± 7.5	8.3 ± 11.8	0.08	0.30
Δ end	15.9 ± 18.5	8.1 ± 9.1	7.1 ± 10	0.014*	0.025*
Δ min	20.5 ± 18.4	13.3 ± 11.1	11.6 ± 10	0.015*	0.021*
% 4th	14.7 ± 16.9	6.9 ± 8	9.8 ± 12.7	0.08	0.34
% end	17.8 ± 19.6	9.4 ± 10.2	7.9 ± 11.4	0.010*	0.019*
% min	23.6 ± 19.1	15.9 ± 11.1	13.4 ± 11.2	0.009*	0.012*

Data are provided as means and standard deviations for all three groups: those with recognised complaints, with unrecognised complaints and those without complaints. *P* values are shown for two combinations of the three groups: first for those with recognised complaints versus the other two groups combined, and then for those with any complaint, recognised or not, versus no complaints

Delta: difference between pre-tilt values and values during tilt (positive values indicate a BP fall). Percentages indicate the BP fall as a percentage of pre-tilt baseline values. *P* values denote the *t* test

*Indicates a statistically significant difference (*p* < 0.05)

*DBP* diastolic blood pressure,* MAP* mean arterial pressure,* SBP* systolic blood pressure

Table 2 also shows* p* values for the exploratory alternate bundling resulting in any complaint versus no complaints or no recognition; the results followed the same general pattern in that absolute BP before or during tilt did not differ but BP fell more in the group with complaints.

**Fig. 1:**
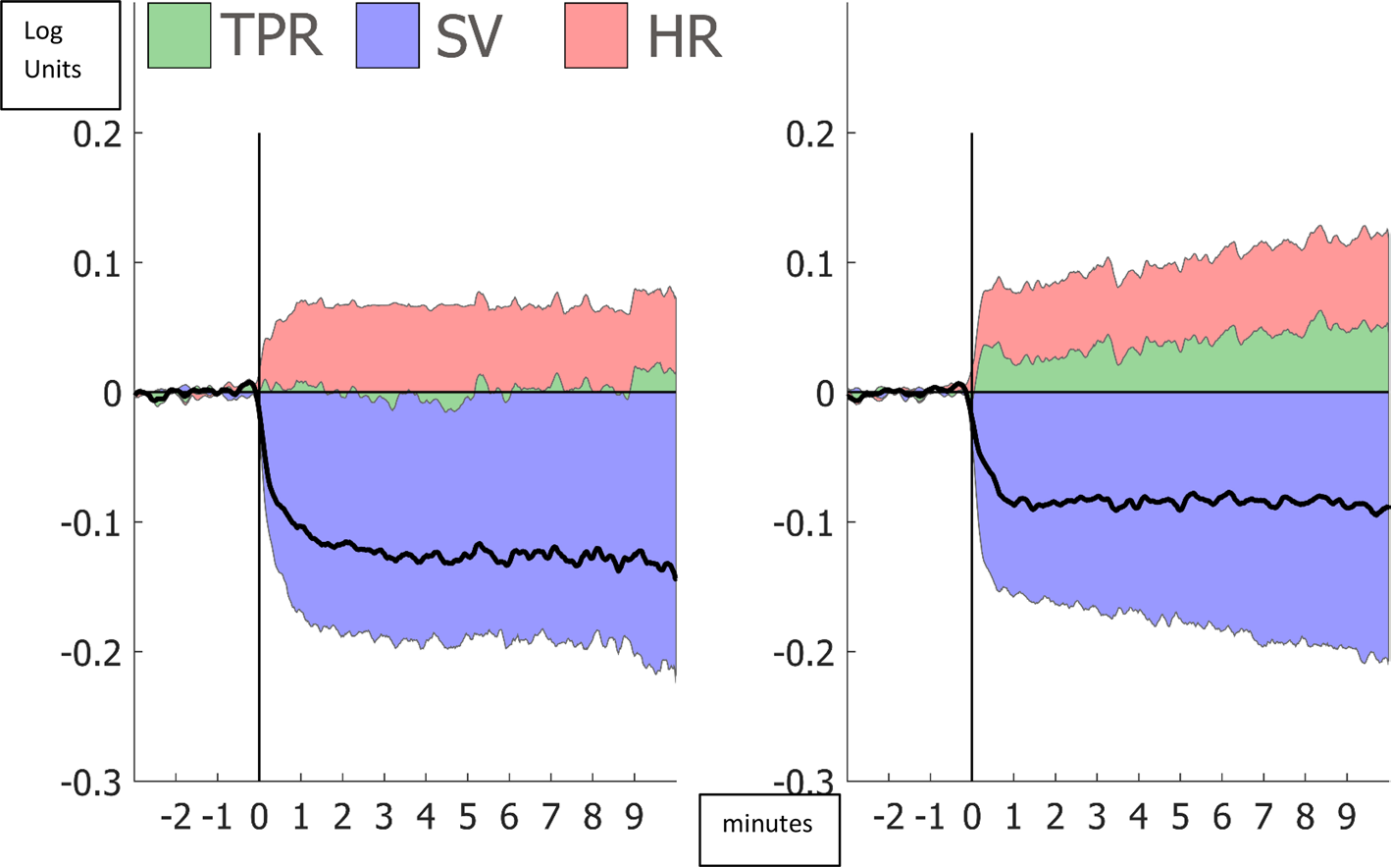
Comparison between group with recognised complaints (left) and group unrecognised complaints or no complaints (right). Cumulative log-ratios during head-up tilt. The x-axis represents time in minutes with time zero as the completion of the act of head-up tilt. Values per parameter represent group log-ratio means. Positive values were stacked starting at zero and negative ones were similarly added, shown downwards. MAP_LR_ is the sum of all positive and negative influences. MAP, TPR, SV and HR are represented as log-ratio units. MAP_LR_, mean arterial pressure (black line); HR_LR_, heart rate (red area); SV_LR_, stroke volume (blue area); TPR_LR_, total peripheral resistance (green area)

Figure 1 and Table 3 show log-ratios for the two groups, bundled as those with recognised complaints versus those with either unrecognised complaints or no complaints. The log-ratio MAP values were lower in the first group for all three periods during tilt; log-ratio TPR values were lower in the first group for the fourth minute and the minimum value, with a trend for the end of tilt. The log-ratios of HR and SV did not differ between the groups.

**Table 3:** log-ratio values of haemodynamic parameters

	Group with recognised complaints (1) Median (IQR)	Group without complaints or recognition (2&3) Median (IQR)	P-values
Fourth minute			
MAP (mmHg)	−0.114 (−0.168 to −0.048)	−0.059 (−0.101 to −0.046)	**0.037**
HR (bpm)	0.058 (0.028 to 0.095)	0.056 (0.026 to 0.082)	0.585
SV (ml)	−0.179 (−0.215 to −0.116)	−0.173 (−0.214 to −0.138)	0.858
TPR (mmHg x sec/ml)	−0.006 (−0.055 to 0.054)	0.0497 (−0.001 to 0.083)	**0.029**
End of TTT			
MAP (mmHg)	−0.128 (−0.245 to −0.072)	−0.086 (−0.122 to −0.055)	**0.011**
HR (bpm)	0.057 (0.007 to 0.116)	0.059 (0.034 to 0.103)	0.564
SV (ml)	−0.199 (−0.261 to −0.143)	−0.199 (−0.265 to −0.150)	0.996
TPR (mmHg x sec/ml)	0.018 (−0.059 to 0.075)	0.055 (0.003 to 0.101)	0.056
Minimum value			
MAP (mmHg)	−0.148 (−0.274 to −0.111)	−0.113 (−0.149 to −0.079)	**0.011**
HR (bpm)	0.037 (−0.002 to 0.107)	0.051 (0.023 to 0.095)	0.472
SV (ml)	−0.267 (−0.357 to −0.184)	−0.271 (−0.307 to −0.197)	0.826
TPR (mmHg x sec/ml)	−0.032 (−0.095 to 0.030)	0.022 (−0.036 to 0.067)	**0.031**

Data are shown as median and interquartile log-ratio values

*P* values denote the Mann–Whitney test

*Indicates a statistically significant difference (*p* < 0.05)

*HR* heart rate, *MAP* mean arterial pressure, *SBP* systolic blood pressure, *SV* stroke volume, *TPR* total peripheral resistance, *TTT* tilt table test

In short, absolute BP before and during tilt did not depend on complaints, but the BP fall was larger for those with complaint, regardless of when BP was measured during tilt, whether the BP change was expressed as a difference of a percentile fall or whether the group with unrecognised complaints was bundled with those with recognised complaints or with those without complaints.

## Discussion

This study investigated the relation of the presence of complaints to changes of BP and its constituent factors during TTT in patients with cOH. The main finding was that minimum SBP, MAP and to a lesser extent DBP during tilt did not differ according to the presence of complaints, but the BP fall did: BP decreased more in those with recognised complaints than in those with no complaints or unrecognised complaints. The same pattern held when a group with any complaint, recognised or not, was compared with those without any complaint. As the group with unrecognised complaints was small (*n* = 11) its contribution many have not been important. The larger BP decrease of MAP in the group with recognised complaints was accompanied by lower TPR, strongly suggesting that the lower MAP in this group was due to a marked inability to increase TPR in the upright condition. As such, this suggests a strong neurogenic contribution in our entire cOH group [15, 19].

### Comparison with studies on the relation between cOH and complaints

Three earlier major studies regarding BP and symptom occurrence in OH yielded different results. Arbogast et al. examined the presence and types of symptoms in patients with OH who experienced a SBP drop greater than 60 mmHg. They found that most patients with profound orthostatic hypotension lack typical symptoms during blood pressure drops. About one quarter experience only atypical symptoms, and one third have no symptoms at all despite similar clinical features. According to this study, this lack of awareness likely reflects dysfunction in neural signalling or central processing, develops over time, and significantly increases health risk due to the absence of protective symptoms [12]. Palma et al. reported that complaints were related to low absolute upright BP, but not to the BP fall, with high sensitivity and specificity in a group of patients with PD. Freeman et al. found no correlation of symptoms with either low absolute BP or the magnitude of BP decline in patients with different neurological disorders [11, 13]. Our study resulted in yet another pattern: we found a relation with the BP fall but not with absolute BP in a population of patients with cOH and various neurological disorders.

The reasons for these variable results are unclear. One possible explanation lies in different study populations. Our population did not only comprise participants with PD as studied by Palma et al. [11]. Instead, our population more resembled the one studied by Freeman et al., who included participants with various neurological diagnoses, ranging from alpha-synucleinopathies to diabetic neuropathy. A second explanation might be how complaints were assessed. Whereas Freeman et al. distinguished three specific symptoms, i.e. light-headedness, dizziness and blackouts [13], and Arbogast et al. categorised the complaints in typical, atypical and asymptomatic [12], we opted to categorise symptoms based on recognition of complaints during TTT as similar to ones in daily life, in line with the recommendations of the European Federation Of Autonomic Societies [2]. We stress that the mere presence or absence of complaints does not fully capture the severity of BP changes, yet complaints alone drive patients to visit outpatient clinics.

Unfortunately, the reasons for the differences in outcome remain uncertain and suggest that a detailed comparison of tilt procedures might ascertain unidentified differences.

### Pathophysiology of complaints in cOH

The discrepancies in the relation between BP and symptom recognition across studies suggest a complex pathophysiology, affecting all links in a chain from low blood pressure to perception of complaints in cOH.

The rate of BP decline may influence symptom development. In cOH, BP can drop slowly or rapidly. Extremely rapid drops, such as those seen in sudden asystole, may cause symptoms to go unreported [20]. In cOH, the decline is generally slower than in asystole, though the rate can still vary considerably. When BP decreases very slowly, compensatory mechanisms, including cerebral autoregulation, have time to act, reducing symptoms related to cerebral hypoperfusion. When BP falls more rapidly, but not as fast as in asystole, patients are more likely to notice and report symptoms. The frequency of cOH episodes may also influence symptom development, as patients with neurogenic causes of cOH often develop long-term adaptations to maintain cerebral perfusion.

The only safe assumption about the relation between absolute BP and cOH is that cerebral hypoperfusion must occur if BP becomes low enough, ultimately leading to syncope.

### Role of cerebral autoregulation

Although the brain accounts for only 2–3% of body weight, it receives about 15–20% of cardiac output [21]. Syncope results from a short-term decrease in cerebral blood flow (CBF), lasting long enough to deplete the brain’s energy reserves, where neuronal damage can start within 2 min of ischaemia. CBF is regulated by four key mechanisms: autoregulation, chemoregulation, neurovascular control and endothelial-dependent factors [20].

Cerebral autoregulation is primarily effective in responding to gradual changes in blood pressure (BP) over hours, days or longer, and is less effective in handling very rapid fluctuations that occur within seconds, such as during most syncope episodes. The ability of cerebral autoregulation to adapt to BP changes is affected by age, chronic illness and autonomic function [20]. Novak et al. described three autoregulatory response patterns in patients with OH, focusing on blood flow velocity and MAP: impaired regulation with a flat flow–BP curve, an extended autoregulated range and failure of the autoregulation with a steep BP curve. In Novak’s study, most patients with OH experienced mild autoregulatory failure and only developed symptoms with significant BP drops. About 25% had severe autoregulatory failure, leading to cerebral hypoperfusion even with small BP changes [22]. These findings illustrate that the severity of BP in cOH does not safely predict the severity of cerebral hypoperfusion: if BP is low but too high to cause syncope, the severity of cOH provides no certainties regarding complaints.

### Cerebral hypoperfusion and cerebral dysfunction as the cause of symptoms

BP is the product of HR, SV and TPR. If any one of these decreases enough, the result is low BP and ultimately cerebral hypoperfusion, depending on the efficiency of CBF autoregulation. The severity and duration of hypoperfusion dictate the degree of brain dysfunction, starting with mild hypoperfusion and subtle cortical dysfunction, e.g. attention deficits. More severe hypoperfusion causes dysfunction and ultimately cessation of first cortical and later brainstem activity [19, 23–25].

However, the presence of complaints requires the ability to perceive complaints and hence requires intact awareness, implying a limited degree of cortical dysfunction with probably no more than subtle alterations of cortical function, most likely affecting attention or other cognitive deficits [26]. Not all complaints in cOH appear to be cognitive in nature, suggesting other pathways than cerebral hypoperfusion.

### Non-cerebral symptoms

In neurogenic OH, systemic low BP means that many organs may suffer from hypoperfusion. For instance, muscle hypoperfusion has been held responsible for coat hanger pain or chest pain in cOH. Some complaints in cOH may be due to hyperventilation. Hyperventilation physiologically causes cerebral vasoconstriction and peripheral vasodilation [15, 23]. In neurogenic cOH, hyperventilation may cause an even more marked decrease of TPR that worsens the BP fall in cOH and may further impair cerebral perfusion [23]. Hyperventilation by itself causes a highly variable range of symptoms, including light-headedness, anxiety as well as symptoms that do not originate in the brain, such as pins and needles sensations that probably reflect peripheral neuronal dysfunction.

### Symptoms of unknown origin

Light-headedness is often regarded as a symptom of cerebral hypoperfusion. It is definitely associated with low BP, but we know of no proof that light-headedness is caused by cerebral dysfunction. It may not be, as the earliest cortical dysfunction in cerebral hypoperfusion appears to affect cognition, and light-headedness does not seem to be cognitive in nature. Perhaps light-headedness is related to a mechanical consequence of low intracranial blood pressure rather than brain dysfunction.

### Perception of dysfunction

Some patients report feeling nothing abnormal at all in cOH, even with very large BP falls; many such patients show no evidence of impaired cortical function, and their EEG is unchanged while they experience complaints [20, 26]. How symptoms of cOH are perceived is largely unknown [13]. The ability to note deficiencies in one’s own degree of attention may well vary considerably between individuals. It is also possible that some complaints rely on interoception, meaning on autonomic afferents that may be damaged in autonomic failure, as recently proposed for Parkinson’s disease [27]. Finally, some patients may not report perceptions they have become accustomed to.

### Limitations

The study was not primarily designed to study the emergence of complaints during the evolution of cOH, which however also held for previous studies [11, 13]. Our study was retrospective which may have hampered the data collection. Although this did not allow us to determine the temporal course of complaints, we could reliably determine the presence or absence of complaints as this was part of our clinical routine. We included drug use at the time of TTT but lack information of timing of intake and duration of medication use. We cannot exclude that drugs affected the findings, but doubt that complaint recognition is specifically affected by medication. A single TTT may not fully capture real-world experiences of OH. Symptoms may not appear if blood pressure does not drop significantly, whereas an asymptomatic blood pressure fall can still yield important clinical information.

## Conclusion

Establishing a clear relation between haemodynamic changes and symptoms in cOH yields variable findings, as indicated by differences between all reported studies. The varying conclusions may partly stem from diverse study populations, but we suspect that there can in fact be no clear relation between low BP and perception of complaints, because there are too many modifying steps between the two. Cerebral autoregulation is probably the major modifier, meaning that low BP does not equal cerebral hypoperfusion, except when BP is so low that autoregulation does not work or syncope occurs. Assessing the nature of complaints in cOH may require determining all steps between low BP and symptoms, starting with cerebral hypoperfusion and brain function.

## Data Availability

Available upon reasonable request.

## Abbreviations

BP: Blood pressure
CBF: Cerebral blood flow
cOH: Classical orthostatic hypotension
DBP: Diastolic blood pressure
HR: Heart rate
LR: log-ratio
MAP: Mean arterial pressure
SBP: Systolic blood pressure
SV: Stroke volume
TPR: Total peripheral resistance
TTT: Tilt table test
